# Minimally Invasive Intramedullary Fixation vs Plate Fixation of Distal Fibular Fractures in Elderly Patients: A Natural Experiment

**DOI:** 10.1177/10711007261427486

**Published:** 2026-04-16

**Authors:** David B. Tas, Diederik P. J. Smeeing, Egbert Jan M. M. Verleisdonk, Jort Keizer, Detlef van der Velde, Roderick M. Houwert, Benjamin L. Emmink

**Affiliations:** 1 Department of Trauma Surgery, University Medical Center Utrecht, Utrecht, the Netherlands; 2 Department of Trauma Surgery, Diakonessenhuis, Utrecht, the Netherlands; 3 Department of Trauma Surgery, Antonius Hospital, Utrecht, the Netherlands; 4 Department of Trauma Surgery, Rijnstate Hospital, Arnhem, the Netherlands; 5 Utrecht Traumacenter, Utrecht, the Netherlands; 6 Department of Trauma Surgery, Gelre Hospital, Apeldoorn, the Netherlands

**Keywords:** fibula, fracture, ankle, intramedullary fixation, nail, elderly, natural experiment

## Abstract

**Background:**

The aim of this study was to compare the postoperative complications and functional outcomes of intramedullary fixation (IMF) and conventional plate fixation (PF) for Lauge-Hansen supination external rotation type 4 fractures in patients aged 70 years or older.

**Methods:**

This natural experiment involved 2 hospitals (A and B) in a single trauma region. Between June 2019 and January 2023, a total of 80 patients aged 70 years or older with an unstable supination external rotation type 4 fracture were treated with either a geriatric treatment pathway consisting of early-stage IMF following immediate protected full weight bearing in hospital A (n = 48) or PF using a one-third tubular locking plate in hospital B (n = 32). The primary outcome was the total number of postoperative complications during 12 months of follow-up.

**Results:**

The IMF group had a higher mean age compared with the PF group (79.8 vs 75.7 years, *p* < .001). The total number of postoperative complications was lower in the IMF group compared with the PF group (27% vs 58%, *p* = .030). Wound infections were observed less frequently following IMF compared to PF (15% vs 35%, *p* = .031), with infection treatment involving no implant removal in the IMF group (0/7 for IMF vs 4/11 for PF). Intramedullary fixation resulted in better functional outcome measured by the Olerud-Molander Ankle Score (*p* = .040) and less postoperative pain (*p* < .001) compared to PF, with the greatest relative differences observed at 6 weeks postoperatively.

**Conclusion:**

Intramedullary fixation resulted in significantly less postoperative complications and better functional outcome compared to PF, despite the higher mean age of patients in the IMF group. A geriatric treatment pathway using an intramedullary nailing technique may be preferred over conventional PF in an elderly population, especially in case of patient obesity or compromised general health status.

**Level of Evidence:** Level II, prospective cohort study.

## Introduction

Ankle fractures are common in elderly patients, with an incidence of 164 fractures per 100 000 people each year in Europe.^1,2^ This incidence is currently increasing with the advancing age of the population, showing a higher number of Lauge-Hansen supination external rotation type 4 fibular fractures compared with more stable types of ankle fractures.^1,3^

Surgical fixation is preferred over nonoperative treatment for unstable ankle fractures, for which the current standard is plate fixation (PF).^3^ Although open reduction and internal PF allows anatomical restoration of the ankle joint, it is associated with a considerable risk of wound-related complications and implant failure in elderly patients and patients with chronic comorbidity.^4-6^

Intramedullary fixation (IMF) has been described as a minimally invasive alternative to PF. Compared with non-anatomic PF, IMF uses smaller incisions and a lower-profile implant, providing superior biomechanical strength and allowing for surgery in an earlier stage after trauma regardless of soft tissue swelling.^6,7^ As a potential result, IMF is associated with fewer wound-related complications and implant removals compared with PF.^5,8,9^ Furthermore, the superior biomechanical strength of the intramedullary construct allows for immediate protected full weight bearing after IMF, potentially leading to shorter duration of hospital stay and better preservation of ankle function. Especially in a fragile elderly population with osteoporotic bone and compromised soft tissue, these advantages of IMF may outweigh the benefit from optimal fracture reduction in PF.

Previous studies have compared an intramedullary nailing technique vs conventional PF in an elderly population.^10-16^ In contrast, this study established a clinical pathway for treatment of unstable ankle fractures in elderly patients, consisting of early intramedullary nail fixation and immediate postoperative mobilization in a walking cast. Therefore, this study investigated a geriatric treatment pathway using an intramedullary nailing technique rather than a surgical technique alone.

The aim of this natural experiment is to compare the postoperative complications and functional outcomes of IMF and conventional PF for Lauge-Hansen supination external rotation type 4 fractures in patients aged 70 years or older. The study hypothesized that a geriatric treatment pathway using IMF, when compared to conventional PF, would result in less postoperative complications by allowing a minimally invasive technique, while providing superior preservation of ankle function as surgery can be performed in an earlier stage after trauma with postoperative treatment allowing immediate protected full weight bearing.

## Methods

This study was performed according to the Strengthening the Reporting of Observational Studies in Epidemiology (STROBE) guidelines.^17^ The study protocol was registered in the Dutch Trial Register (NL-OMON20210, July 2019).

### Study Design

This natural experiment involved two hospitals in a single trauma region in the Netherlands, including the St Antonius Hospital, Nieuwegein (hospital A); and the Diakonessenhuis, Utrecht (hospital B). Both hospitals are level 2 trauma centers. Between June 2019 and January 2023, a total of 80 patients aged 70 years or older with an unstable Lauge-Hansen supination external rotation type 4 fracture was included in this study. Patients were treated according to the standard protocol of the involved hospital. Patients for the IMF group were recruited from hospital A, whereas patients for the PF group were recruited from hospital B. Therefore, treatment allocation was based on the patient’s location at the time of presentation, independent of the investigators’ control.^18^ All patients were consecutively recruited from the emergency room or outdoor department of the participating hospitals. Fracture classification according to the Lauge-Hansen classification system was performed by an expert panel consisting of 7 experienced trauma surgeons.^19^

Ethical approval was granted by the Medical Ethics Committee of the participating hospitals (W19.025, February 2019).

### Patient Population

The inclusion criteria were all patients aged 70 years or older with a fracture classified as Lauge-Hansen supination external rotation type 4 including luxation fractures. The threshold of 70 years for inclusion of a geriatric patient population was based on a risk factor for development of postoperative complications after ankle surgery.^20^ Medial instability in Lauge-Hansen supination external rotation type 4 fractures was defined as either a radiographically confirmed medial malleolar fracture, a medial clear space of more than 4 mm with that value exceeding the superior tibiotalar displacement by 1 mm, or a deep deltoid ligament lesion confirmed by diagnostic imaging.^21^

The exclusion criteria were (1) pathologic fractures, (2) severely comminuted fractures, (3) fractures older than 14 days, (4) patients involved in polytrauma, and (5) inoperable patients. Fractures older than 14 days were excluded because fracture callus impedes percutaneous fracture reduction, posing a contraindication to a minimally invasive IMF technique.^6^ Polytrauma is defined as significant injuries in 2 or more different anatomic regions associated with at least 3 points on the Abbreviated Injury Scale (AIS) or with an Injury Severity Score (ISS) of at least 15 points.^22^ Inoperable patients included patients with contraindications to anesthesia or an intramedullary technique, such as preinjury deformity or narrowing of the fibular medullary canal to less than 3.1 mm.^23^

Baseline characteristics with corresponding definitions are provided in Appendix 1.

### Intervention

Patients in the IMF group were scheduled for surgery within 48 hours after presentation if possible.^24^ Intramedullary fixation was performed with the Acumed Fibular Rod System (Hillsboro, OR). The nail was inserted using a 1- to 2-cm longitudinal incision at 1 cm distal to the tip of the fibula. Once fracture reduction was achieved, the nailing construct was locked using 1 or 2 distal anterior-to-posterior 3.5-mm headless cortical screws and 1 or 2 proximal transsyndesmotic 3.5-mm headless cortical screws. Postoperative treatment of IMF consisted of a below-knee circular cast for 6 weeks, allowing immediate protected full weight bearing. Cast replacement was performed 7-14 days postoperatively, allowing wound inspection.

Patients in the PF group were scheduled for delayed-stage surgery in order to allow soft tissue swelling to subside and be amenable for surgery. Open reduction and internal fixation was performed according to the Arbeitsgemeinschaft für Osteosynthesefragen (AO) Foundation principles of fracture fixation.^25^ Plate fixation was performed using a 3.5-mm non-anatomical one-third tubular locking plate and 3.5mm headed screws (DePuy Synthes, Oberdorf, Switzerland) according to standard treatment protocol of the involved hospital. Syndesmosis fixation was performed using 1 or 2 transsyndesmotic 3.5-mm screws based on preoperative testing and surgeon’s preference. Postoperative treatment of PF consisted of non–weight bearing for 6 weeks, as is common in patients aged >70 years with a supination external rotation type 4 ankle fracture.^26^ Unprotected postoperative treatment allowed repeated wound inspection and range of motion exercises of the ankle.

### Outcome Measures

The study outcomes and corresponding definitions are provided in Appendix 2.

The primary outcome measure of this study was the total number of postoperative complications, including (1) wound infection, (2) wound healing disorders, (3) implant-related complications, (4) deep vein thrombosis, (5) pulmonary embolism, and (6) mortality. Implant-related complications included re-operation due to a symptomatic implant, implant failure, and inadequate fixation.

Secondary outcome measures were the Olerud-Molander Ankle Score (OMAS), the Parker Mobility Score, the visual analogue scale (VAS) for pain, duration of hospital stay, and number of postoperative hospital visits. The OMAS is a validated questionnaire for functional objective outcome with a score ranging from 0 to 100 (Appendix 3).^27^ The Parker Mobility Score evaluates the walking ability with a score ranging from 0 to 9, predicting patient mortality (Appendix 4).^28^

### Follow-up

A total follow-up time of 12 months was chosen in this study, which is in accordance with previous studies evaluating the complications after IMF of ankle fractures.^10,12,14^ All patients were reviewed in the outpatient department by the treating surgeon and/or investigator at 2 weeks, 6 weeks, 3 months, and 12 months postoperatively. Patient visits consisted of a standardized clinical examination, including wound inspection and assessment of the functional outcome scores. The study data were collected and inserted into the study database by a single investigator (DT).

### Statistical Methods

Statistical analysis was conducted according to the intention-to-treat principle. Dichotomous variables were analyzed using the Pearson χ^2^ test (all cells ≥5) or Fisher exact test (1 or more cells <5). Continuous variables were analyzed using Student *t* test (normally distributed data) or Mann-Whitney *U* test (non–normally distributed data) and presented as means with SDs and 95% CIs. A linear mixed model was used for the OMAS, Parker Mobility, and VAS scores to account for the repeated measures during follow-up. Subgroup analyses of the primary study outcome were performed for patient age (80 years or older), body mass index (above 30), American Association of Anesthesiologists classification (ASA class III-IV), smoking, presence of comorbidities, and time between trauma and surgery (longer than 48 hours).^29^ Subgroup analyses were presented descriptively, as limited sample sizes precluded formal statistical testing for significance. A *P* value of less than .05 was considered statistically significant in all analyses. Statistical analyses will be performed using the SPSS software, version 25.0 (IBM Corp).

## Results

### Patient Population

**Figure 1.**
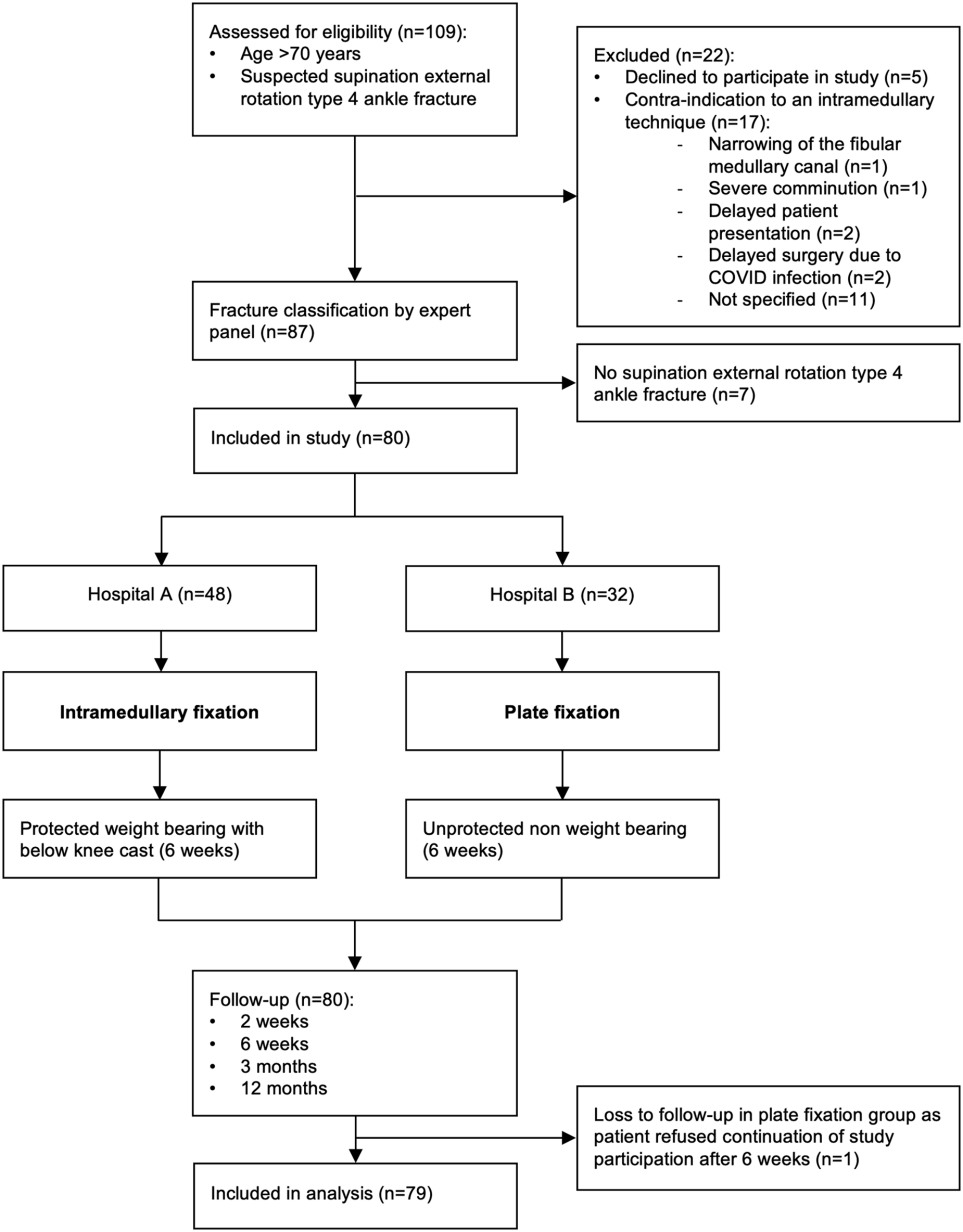
Flow diagram of a natural experiment on minimally invasive intramedullary fixation vs plate fixation of distal fibular fractures in elderly patients. Hospital A: St Antonius Hospital, Nieuwegein; Hospital B: Diakonessenhuis, Utrecht.

A flow diagram of the study is provided in Figure 1. A total of 80 patients were included. One patient in the PF group refused continuation of study participation after 6 weeks. Study analysis was performed on a total of 79 patients (48 patients in the IMF group and 31 patients in the PF group).

The baseline characteristics are provided in Table 1. The mean age of the total population was 78.2 years (range 70-94). Patients in the IMF group had a statistically significantly higher mean age (79.8 ± 6.1 years) compared with the PF group (75.7 ± 5.2 years, *P* < .001). No deltoid ligament repair was performed.

**Table 1.** Baseline Characteristics of a Natural Experiment on Minimally Invasive Intramedullary Fixation vs Plate Fixation of Distal Fibular Fractures in Elderly Patients.

Characteristics	Total Population	IMF	PF	*P* Value^a^
Patient demographics				
Patients included, n	79	48	31	
Age, y, mean ± SD (95% CI)	78.2 ± 6.1 (76.8-79.5)	79.8 ± 6.1 (78.0-81.6)	75.7 ± 5.2 (73.6-77.5)	**<.001**
Female sex, n (%)	64 (81)	40 (83)	24 (77)	.513
BMI, mean ± SD (95% CI)	28.0 ± 4.9 (26.9-29.1)	28.6 ± 5.1 (27.1-30.1)	27.1 ± 4.4 (25.5-28.7)	.192
ASA classification, n (%)				.588
I	8 (10)	6 (13)	2 (7)	
II	44 (56)	25 (52)	19 (61)	
III	27 (34)	17 (35)	10 (32)	
IV	0	0	0	
Smoking, n (%)	6 (8)	2 (4)	4 (13)	.204
Co-morbidities, n (%)				
Compromised local soft tissue	21 (27)	15 (31)	6 (19)	.243
Diabetes mellitus	15 (19)	10 (21)	5 (16)	.603
Peripheral arterial disease	7 (9)	4 (8)	3 (10)	1.000
Venous insufficiency	3 (4)	3 (6)	0	.276
Psychiatric disease	10 (13)	3 (6)	7 (23)	.**043**
Pre-existent immobility	1 (1)	0	1 (3)	.392
Preinjury OMAS, mean ± SD (95% CI)	82.9 ± 15.1 (79.5-87.7)	80.5 ± 15.1 (75.5-85.6)	86.6 ± 14.5 (80.2-93.0)	.085
Preinjury Parker Mobility Score, mean ± SD (95% CI)	7.8 ± 1.9 (7.3-8.3)	7.7 ± 1.8 (7.0-8.3)	7.9 ± 1.9 (7.2-8.7)	.400
Trauma demographics				
Side of injury: left, n (%)	39 (49)	24 (50)	15 (48)	.889
Trauma mechanism, n (%)				.502
Simple supination external rotation trauma	69 (87)	43 (90)	26 (84)	
Traffic accident	10 (13)	5 (10)	5 (16)	
Time between trauma and surgery, d, mean ± SD (95% CI)	6.4 ± 5.7 (5.2-7.7)	3.6 ± 4.1 (2.4-4.8)	10.8 ± 5.2 (8.9-12.7)	**<.001**
Fracture demographics				
Malleolar involvement, n (%)				.387
Unimalleolar	12 (15)	9 (19)	3 (10)	
Bimalleolar	8 (23)	9 (19)	9 (29)	
Trimalleolar	49 (62)	30 (63)	19 (61)	
Luxation fracture, n (%)	46 (58)	29 (60)	17 (55)	.624
Comminuted fracture, n (%)	34 (43)	23 (48)	11 (36)	.276
Open fracture, n (%)	12 (15)	7 (15)	5 (16)	1.000
Gustilo I	2 (3)	1 (2)	1 (3)	
Gustilo II	8 (10)	4 (8)	4 (13)	
Gustilo III	2 (3)	2 (4)	0	

Abbreviations: ASA, American Association of Anesthesiologists; BMI, body mass index; IMF, intramedullary fixation; OMAS, Olerud Molander Ankle Score; PF, plate fixation.

a Boldface indicates significance (*P* < .05).

A post hoc power analysis was performed based on the primary outcome measure of this study. A minimal clinically relevant difference of 31% was derived from the total postoperative complication rates observed in this study (27% for IMF and 58% for PF). A 2-sided test with an *α* = .05 and *β* = .20 resulted in a required sample size of 72 patients, confirming that this study was adequately powered (80%) to detect the specified effect size for the primary outcome.

### Postoperative Complications

The postoperative complication rates were high in both treatment groups. However, the total number of postoperative complications was significantly lower in the IMF group (n = 13, 27%) compared with the PF group (n = 18, 58%, *P* = .030) (Table 2). This difference was predominantly explained by a significantly lower number of wound infections following IMF (n = 7, 15%) compared with PF (n = 11, 35%, *P* = .031). Wound infection demanding reoperation was not observed in the IMF group (0 of 7), whereas 4 patients with a wound infection in the PF group were treated with plate removal (4 of 11, 36%). Wound healing disorders were similar for IMF and PF (6% vs 3%, *P* = 1.000), with all patients showing delayed wound healing (more than 4 weeks).

**Table 2.** Postoperative Complications of a Natural Experiment on Minimally Invasive Intramedullary Fixation vs Plate Fixation of Distal Fibular Fractures in Elderly Patients.^a^

Outcomes	IMF, n (%) (n = 48)	PF, n (%) (n = 31)	*P* Value^b^
Total number of postoperative complications	13 (27)	18 (58)	.**030**
Wound infection	7 (15)	11 (35)	.**031**
Treated with antibiotics	7 (15)	7 (23)	
Treated with re-operation	0	4 (13)	
Wound healing disorders	3 (6)	1 (3)	1.000
Implant-related complications	2 (4)	5 (16)	.105
Symptomatic implant	1 (2)	3 (10)	
Implant failure	0	1 (3)	
Inadequate fixation	1 (2)	1 (3)	
Deep venous thrombosis	0	0	NC
Pulmonary embolism	0	0	NC
Mortality	1 (2)	1 (3)	1.000

Abbreviations: IMF, intramedullary fixation; NC, not calculable; PF, plate fixation.

a Decision making regarding postoperative complications (such as the threshold for re-operation in cases of wound infection) may differ between treatment teams of the hospitals involved in this natural experiment.

b Boldface indicates significance (*P* < .05).

Implant-related complications were observed less frequently after IMF (n = 2, 4%) compared with PF (n = 5, 16%). Implant failure was not observed following IMF, whereas 1 patient in the PF group demonstrated breakage of the plate 2 weeks postoperatively, requiring implant removal and definitive treatment using an external fixation device.

In each study group, 1 patient died during follow-up (at 8 months in the IMF group and 7 months in the PF group) with no causal relationship to the study treatment.

### Functional and Hospitalization Outcomes

Mixed model analyses of repeated measures of the OMAS at 6 weeks, 3 months, and 12 months revealed better functional outcome following IMF compared with PF (*P* = .040) (Table 3). Postoperative pain assessed by the VAS was less in the IMF group compared with the PF group over time (*P* < .001). The greatest between-group differences in functional outcome scores were observed in short-term assessment at 6 weeks postoperatively (Table 3).

**Table 3.** Functional Outcome Scores and Hospitalization Outcomes of a Natural Experiment on Minimally Invasive Intramedullary Fixation vs Plate Fixation of Distal Fibular Fractures in Elderly Patients.

Outcomes	IMF (n = 48)	PF (n = 31)	*P* Value^a^
OMAS (range 0-100), mean ± SD (95% CI)			.**040**^b^
Preinjury	80.5 ± 15.1 (75.5 to 85.6)	86.6 ± 14.5 (80.2 to 93.0)	
6 wk	44.0 ± 16.9 (38.8 to 49.1)	27.6 ± 16.4 (21.2 to 34.0)	
3 mo	58.9 ± 17.4 (53.7 to 64.0)	50.0 ± 20.5 (43.6 to 56.4)	
12 mo	67.6 ± 20.4 (62.2 to 72.6)	60.0 ± 22.6 (53.8 to 66.7)	
Parker Mobility Score (range 0-9), mean ± SD (95% CI)			.692^b^
Preinjury	7.7 ± 1.8 (7.0 to 8.3)	7.9 ± 1.9 (7.2 to 8.7)	
6 wk	5.5 ± 2.3 (4.8 to 6.1)	4.0 ± 2.0 (3.2 to 4.8)	
3 mo	6.6 ± 2.1 (6.0 to 7.2)	6.7 ± 2.0 (5.9 to 7.4)	
12 mo	6.6 ± 2.6 (6.0 to 7.2)	7.1 ± 2.6 (6.3 to 7.9)	
VAS for pain (range 0-100), mean ± SD (95% CI)			**<.001** ^b^
2 wk	33.2 ± 24.9 (26.3 to 40.0)	44.0 ± 32.5 (35.4 to 52.5)	
6 wk	23.7 ± 24.1 (16.8 to 30.6)	44.1 ± 26.8 (35.6 to 52.7)	
3 mo	13.2 ± 20.7 (6.3 to 20.1)	32.7 ± 27.2 (24.1 to 41.2)	
12 mo	5.9 ± 14.6 (–1.1 to 12.7)	19.4 ± 24.2 (10.6 to 27.9)	
Duration of hospital stay, d, mean ± SD (95% CI)	5.8 ± 4.9 (4.4 to 7.2)	7.5 ± 8.2 (4.5 to 10.5)	.972
Number of postoperative hospital visits, mean ± SD (95% CI)	4.5 ± 2.7 (3.7 to 5.2)	6.5 ± 4.4 (4.8 to 8.1)	.**025**

Abbreviations: IMF, intramedullary fixation; OMAS, Olerud Molander Ankle Score; PF, plate fixation; VAS, visual analogue scale.

a Boldface indicates significance (*P* < .05).

b Mixed model analyses on repeated measures of functional outcome scores for differences between intramedullary fixation and plate fixation.

The mean duration of hospital stay was 5.8 ± 4.9 days in the IMF group and 7.5 ± 8.2 days in the PF group (*P* = .972). The mean number of postoperative hospital visits following IMF was lower compared to PF (4.5 ± 2.7 vs 6.5 ± 4.4, *P* = .025).

### Subgroup Analyses

The greatest differences in postoperative complication rates favoring IMF over PF were observed in the subgroups for patient age >80 years (37% for IMF and 71% for PF), BMI >30 (31% for IMF and 100% for PF), and ASA class III or IV (41% for IMF and 90% for PF) (Table 4). In these subgroups, the benefit of reduced postoperative complications associated with IMF may be the greatest.

**Table 4.** Subgroup Analysis of the Total Number of Postoperative Complications Observed in a Natural Experiment on Minimally Invasive Intramedullary Fixation vs Plate Fixation of Distal Fibular Fractures in Elderly Patients.

Subgroups	Total Number of Postoperative Complications	
	IMF, n/n (%)	PF, n/n (%)
Total population (n = 79)	13/48 (27)	18/31 (58)
Age >80 y (n = 26)	7/19 (37)	5/7 (71)
BMI >30 (n = 25)	5/16 (31)	9/9 (100)
ASA classification III-IV (n = 27)	7/17 (41)	9/10 (90)
Smoking (n = 6)	0/2 (0)	4/4 (100)
Presence of comorbidities:		
Compromised local soft tissue (n = 21)	6/15 (40)	5/6 (83)
Diabetes mellitus (n = 15)	6/10 (60)	5/5 (100)
Peripheral arterial disease (n = 7)	3/4 (75)	1/3 (33)
Venous insufficiency (n = 3)	2/3 (67)	0/0
Psychiatric disease (n = 10)	1/3 (33)	4/7 (57)
Pre-existent immobility (n = 1)	0/0	0/1
Time between trauma and surgery >48 hours (n = 48)	2/18 (11)	18/30 (60)

Abbreviations: ASA, American Association of Anesthesiologists; BMI, body mass index; IMF, intramedullary fixation; PF, plate fixation.

## Discussion

This natural experiment included a total of 80 patients who were treated with either IMF or conventional PF in a single trauma region in the Netherlands. Although complication rates were high in both treatment groups, IMF resulted in a significantly lower number of postoperative complications, with complication treatment involving less re-operation compared with PF. However, the study design limits the ability to isolate the effect of the surgical treatment from other systematic differences between hospitals.

Five previous studies have compared IMF using a similar locking fibular nailing system to conventional PF in an elderly population.^12-16^ The postoperative complication rates observed in this study (27% for IMF and 58% for PF) were high when compared to these previous studies (range 12%-27% for IMF and 18%-59% for PF). This difference in postoperative complication rates may be explained by the relatively fragile geriatric patient population included in this study, showing a higher mean age (78 years vs 70-74 years in previous studies) and inclusion of patients with pre-injury immobility, cognitive impairment, or inability to comply with postoperative regime. Additionally, this study included a higher number of open fractures compared to previous studies (15% vs 0%-5% respectively), reflecting the fragile geriatric population included in this study.^12-14,16^ Finally, this study evaluated wound healing disorders as a postoperative complication, with all cases presenting as delayed wound healing. Although providing a more comprehensive assessment of wound-related disorders, incorporation of this outcome may have contributed to the higher number of postoperative complications observed in this study relative to previous studies that restricted their evaluation to wound infection.

Functional outcome has been described to be similar for IMF and PF in elderly patients, with studies by White et al^12^ and Stake et al^13,15^ showing no significant differences in mean OMAS at 3, 6, 12, and 24 months of follow-up. Also, no difference in postoperative pain was observed between IMF and PF.^13^ In contrast to these previous studies, this study showed a significant difference in repeated measures of OMAS (*P* = .040) and VAS for postoperative pain (*P* < .001) favoring IMF over PF, with the greatest in-between differences observed at 6 weeks postoperatively. The difference in OMAS observed in this study (67.6 for IMF and 60.0 for PF after 12 months follow-up) can be regarded as clinically relevant, considering a minimal clinically important difference of 7.5 points.^30^ The superior functional outcome observed in this study may be explained by the implementation of a geriatric treatment pathway for IMF, allowing a shorter time to definitive surgery, shorter duration of hospital stay, and immediate mobilization with protected weight bearing following IMF. Especially in an elderly population, the better short-term functional outcome and reduced length of hospital stay represent major advantages of an IMF treatment pathway, potentially outweighing the long-term disadvantage of suboptimal fracture reduction.

Despite the treatment pathway of this study aimed to perform IMF within 48 hours after patient presentation, the mean interval between trauma and surgery was 3.6 days for IMF (and 10.8 days for PF). A delay in patient presentation and the high mean age of patients included in the IMF group may explain the longer interval between trauma and surgery than anticipated in this study. Nevertheless, the shorter time to surgery for IMF may have contributed to the substantially reduced risk of wound infection and necessity for implant removal compared with PF in this study.^31^ Therefore, a shorter time to definitive surgery as part of an IMF treatment pathway may even further decrease the risk of wound infection, necessity of implant removal, duration of hospital stay, and treatment costs in a fragile geriatric patient population.

Although anatomic distal fibular plating is associated with a similar complication rate and functional outcome compared with one-third tubular plating, this technique may be preferred in comminuted or osteoporotic fractures.^32^ As this study included an elderly patient population, the use of an anatomic distal fibular plating technique may have allowed for earlier postoperative weight bearing, potentially leading to better functional outcome in the PF group.

This natural experiment is limited by several potential sources of bias inherent in the study design: (1) patient populations may differ systematically between the involved hospitals based on referral patterns or geographic catchment areas, (2) surgical expertise and technique may vary between institutions and surgeons, (3) decision making regarding complications may differ between treatment teams, and (4) unmeasured differences in perioperative care protocols beyond the specified weight-bearing restrictions may influence outcomes. Although we attempted to minimize selection bias through consecutive enrollment and the geographic proximity of the hospitals within a single trauma region, these unmeasured confounders limit our ability to attribute differences solely to the surgical treatment. Furthermore, the frequency of syndesmotic fixation was not reported and follow-up was limited to 12 months postoperatively. Finally, this study compared two treatment pathways rather than 2 surgical techniques. By evaluating 2 different pathways for treatment of ankle fractures, this study compared not only minimally invasive IMF vs conventional open PF but also early vs late-stage surgery and the different postoperative care regimens associated with the treatment pathways. Although these factors are considered part of the IMF treatment pathway in this study, their potential influence on the study outcomes may impede comparability to alternative studies.

## Conclusion

In this natural experiment including a total of 80 patients, IMF resulted in less postoperative complications and better functional outcome compared to PF for treatment of distal fibular fractures in patients aged 70 years and older. A geriatric treatment pathway using an intramedullary nailing technique allowing for early-stage surgery and immediate postoperative mobilization with protected weight bearing may be preferred over conventional PF in an elderly population, especially in case of patient obesity or compromised general health status.

## Supplemental Material

sj-docx-1-fai-10.1177_10711007261427486 – Supplemental material for Minimally Invasive Intramedullary Fixation vs Plate Fixation of Distal Fibular Fractures in Elderly Patients: A Natural Experiment Supplemental material, sj-docx-1-fai-10.1177_10711007261427486 for Minimally Invasive Intramedullary Fixation vs Plate Fixation of Distal Fibular Fractures in Elderly Patients: A Natural Experiment by David B. Tas, Diederik P. J. Smeeing, Egbert Jan M. M. Verleisdonk, Jort Keizer, Detlef van der Velde, Roderick M. Houwert and Benjamin L. Emmink in Foot & Ankle International

sj-pdf-2-fai-10.1177_10711007261427486 – Supplemental material for Minimally Invasive Intramedullary Fixation vs Plate Fixation of Distal Fibular Fractures in Elderly Patients: A Natural Experiment Supplemental material, sj-pdf-2-fai-10.1177_10711007261427486 for Minimally Invasive Intramedullary Fixation vs Plate Fixation of Distal Fibular Fractures in Elderly Patients: A Natural Experiment by David B. Tas, Diederik P. J. Smeeing, Egbert Jan M. M. Verleisdonk, Jort Keizer, Detlef van der Velde, Roderick M. Houwert and Benjamin L. Emmink in Foot & Ankle International
