# Supplementary material for: Minimally Invasive Intramedullary Fixation vs Plate Fixation of Distal Fibular Fractures in Elderly Patients: A Natural Experiment
Source: Foot Ankle Int. 2026 Apr 16;47(6):735–43. doi: 10.1177/10711007261427486 (PMC13237191; doi:10.1177/10711007261427486)
Supplement: sj-docx-1-fai-10.1177_10711007261427486 – Supplemental material for Minimally Invasive Intramedullary Fixation vs Plate Fixation of Distal Fibular Fractures in Elderly Patients: A Natural Experiment [file sj-docx-1-fai-10.1177_10711007261427486.docx]

|  | **Characteristics** | | **Definitions** |
| --- | --- | --- | --- |
| **Patient**  **demographics** | Age | | Age of patient at time of injury |
|  | Sex | | Sex of the patient |
|  | BMI | | Weight in kilograms / (length in meters)^2^ |
|  | ASA classification | |  |
|  |  | I |  |
|  |  | II |  |
|  |  | III |  |
|  |  | IV |  |
|  | Smoking | |  |
|  |  | Yes | Patients with current tobacco use |
|  |  | No | Patients that never used tobacco or patients that quit smoking |
|  | Co-morbidities | |  |
|  |  | Compromised local soft tissue | Local swelling, blisters, abrasion and/or wound at the fracture site of the injured ankle, posing a contraindication to immediate open reduction and internal fixation |
|  |  | Diabetes mellitus | Diabetes mellitus type 1 or 2 requiring medical treatment (including oral antidiabetic drugs and insulin injections, excluding dietary measures), with or without the presence of disease related complications |
|  |  | Peripheral arterial disease | Ankle-brachial pressure index <0.9 without the necessity of symptoms |
|  |  | Venous insufficiency | Clinical suspicion of venous insufficiency based on edema, pain relieved by elevation of the leg, varicose and/or crural ulcer with confirmed venous reflux |
|  |  | Psychiatric disease | Current presence or history of psychiatric disorder |
|  |  | Pre-existent immobility | Pre-injury inability to walk with necessary use of crutches or wheelchair, or being bound to a lying position |
| **Trauma**  **demographics** | Side of injury | |  |
|  | Trauma mechanism | |  |
|  |  | Simple supination external rotation trauma |  |
|  |  | Fall >3 meters |  |
|  |  | Traffic accident |  |
|  |  | Sports accident |  |
|  |  | Other |  |
|  |  | Unknown |  |
|  | Time between trauma and surgery | | The number of days between the originating trauma and surgical intervention |
| **Fracture demogr.** | Malleolar involvement | | The number of malleoli on the affected ankle with associated fracture regardless of ligament injury |
|  |  | Unimalleolar |  |
|  |  | Bimalleolar |  |
|  |  | Trimalleolar |  |

**Appendix 1** Definitions of baseline characteristics of a natural experiment on minimally invasive intramedullary fixation versus plate fixation of distal fibular fractures in elderly patients

ASA = American Association of Anesthesiologists

BMI = Body Mass Index

**Appendix 2** Definitions of outcome measures of a natural experiment on minimally invasive intramedullary fixation versus plate fixation of distal fibular fractures in elderly patients

| **Outcome measures** | | | | **Definitions** |
| --- | --- | --- | --- | --- |
| Primary | Total number of postoperative complications | | | The total number of postoperative complications as described in this study, occurring during follow-up (12 months) |
|  |  | Wound infection | | A clinical suspicion of wound infection based on redness, purulent drainage, wound dehiscence and/or fever, in combination with the necessity of antibiotic treatment, wound debridement and/or implant removal |
|  |  | | Treated with antibiotics | Oral or intravenous antibiotic treatment by indication of wound infection |
|  |  | | Treated with re-operation | Re-operation in the operating room secondary to initial treatment, for wound debridement and/or implant removal by the indication of wound infection |
|  |  | Wound healing disorders | | Any deviation in the postoperative course, without the necessity of pharmacological or surgical intervention |
|  |  | Implant related complications | | Re-operation after the initial surgical intervention due to implant related symptoms, implant failure or inadequate fracture fixation, without the indication of wound infection |
|  |  | | Symptomatic implant | Symptoms of irritation caused by prominence of the implant material, which is not the result of malpositioning |
|  |  | | Implant failure | Breakage or bending of the implant material, causing articular discongruity of >2 mm |
|  |  | | Inadequate fixation | Malreduction or malpositioned implant without breakage or bending, causing articular discongruity of >2 mm |
|  |  | Deep venous thrombosis | | A deep venous thrombosis confirmed by ultrasound and treated with an anticoagulant |
|  |  | Pulmonary embolism | | Pulmonary emboli confirmed radiographically and treated with an anticoagulant |
|  |  | Mortality | | Loss of life in the period between surgical intervention and end of follow-up (12 months), without the necessity of a demonstrable correlation with the ankle treatment |
| Secondary | OMAS | | | Functional score based on a questionnaire for ankle specific function (range 0 to 100) |
|  | Parker Mobility Score | | | Functional score based on patient mobility (range 0 to 9) |
|  | VAS for pain | | | Score for pain of the injured ankle based on a scale (0 to 100) |
|  | Duration of hospital stay | | | The number of days that the patient is hospitalized for treatment of the injured ankle, in the period between surgical intervention and end of follow-up (12 months) |
|  | Number of postoperative hospital visits | | | The number of postoperative visits to the outpatient department for treatment of the injured ankle, in the period between surgical intervention and end of follow-up (12 months) |

OMAS = Olerud Molander Ankle Score

VAS = Visual Analogue Scale

**Appendix 3** The Olerud Molander Ankle Score

| **Category** | **Degree** | **Score (0-100)** |
| --- | --- | --- |
| Pain | None  While walking on uneven surface  While walking on even surface outdoors  While walking indoors  Constant and severe | 25  20  10  5  0 |
| Stiffness | None  Stiffness | 10  0 |
| Swelling | None  Only evenings  Constant | 10  5  0 |
| Stair climbing | No problems  Impaired  Impossible | 10  5  0 |
| Running | Possible  Impossible | 5  0 |
| Jumping | Possible  Impossible | 5  0 |
| Squatting | No problems  Impossible | 5  0 |
| Supports | None  Taping or wrapping  Stick or crutches | 10  5  0 |
| Work, activities of daily life | Same as before injury  Loss of tempo  Change to a simple job/part-time work  Severely impaired work capacity | 20  15  10  0 |

**Appendix 4** The Parker Mobility Score

| **Walking ability** | **No difficulty** | **Alone with an assistive device** | **With help from another person** | **Not at all** |
| --- | --- | --- | --- | --- |
| Able to walk inside house | 3 | 2 | 1 | 0 |
| Able to walk outside house | 3 | 2 | 1 | 0 |
| Able to go shopping, to a restaurant, or to visit family | 3 | 2 | 1 | 0 |
